# Comparative Preclinical Evaluation of HER2-Targeting ABD-Fused Affibody^®^ Molecules ^177^Lu-ABY-271 and ^177^Lu-ABY-027: Impact of DOTA Position on ABD Domain

**DOI:** 10.3390/pharmaceutics13060839

**Published:** 2021-06-07

**Authors:** Yongsheng Liu, Anzhelika Vorobyeva, Tianqi Xu, Anna Orlova, Annika Loftenius, Theresa Bengtsson, Per Jonasson, Vladimir Tolmachev, Fredrik Y. Frejd

**Affiliations:** 1 Department of Immunology, Genetics and Pathology, Uppsala University, 751 85 Uppsala, Sweden; yongsheng.liu@igp.uu.se (Y.L.); anzhelika.vorobyeva@igp.uu.se (A.V.); tianqi.xu@igp.uu.se (T.X.); fredrik.frejd@affibody.se (F.Y.F.); 2 Research Centrum for Oncotheranostics, Research School of Chemistry and Applied Biomedical Sciences, Tomsk Polytechnic University, 634050 Tomsk, Russia; anna.orlova@ilk.uu.se; 3 Department of Medicinal Chemistry, Uppsala University, 751 83 Uppsala, Sweden; 4 Affibody AB, 171 65 Solna, Sweden; annika.loftenius@affibody.se (A.L.); theresa.bengtsson@affibody.se (T.B.); per.jonasson@affibody.se (P.J.)

**Keywords:** affibody molecule, albumin binding domain (ABD), ^177^Lu, scaffold protein, radionuclide therapy, SKOV-3 xenograft, biodistribution, DOTA

## Abstract

Radiolabeled Affibody-based targeting agent ^177^Lu-ABY-027, a fusion of an anti-HER2 Affibody molecule with albumin binding domain (ABD) site-specifically labeled at the C-terminus, has demonstrated a promising biodistribution profile in mice; binding of the construct to albumin prevents glomerular filtration and significantly reduces renal uptake. In this study, we tested the hypothesis that site-specific positioning of the chelator at helix 1 of ABD, at a maximum distance from the albumin binding site, would further increase the strength of binding to albumin and decrease the renal uptake. The new construct, ABY-271 with DOTA conjugated at the back of ABD, has been labelled with ^177^Lu. Targeting properties of ^177^Lu-ABY-271 and ^177^Lu-ABY-027 were compared directly. ^177^Lu-ABY-271 specifically accumulated in SKOV-3 xenografts in mice. The tumor uptake of ^177^Lu-ABY-271 exceeded uptake in any other organ 24 h and later after injection. However, the renal uptake of ^177^Lu-ABY-271 was two-fold higher than the uptake of ^177^Lu-ABY-027. Thus, the placement of chelator on helix 1 of ABD does not provide desirable reduction of renal uptake. To conclude, minimal modification of the design of Affibody molecules has a strong effect on biodistribution, which cannot be predicted a priori. This necessitates extensive structure-properties relationship studies to find an optimal design of Affibody-based targeting agents for therapy.

## 1. Introduction

Targeted therapy is a useful option for the treatment of disseminated cancer. During the last decades, impressive progress has been made in targeted radionuclide therapy [1]. The most prominent was the progress made in peptide-based radiotherapeutics. However, the number of validated targets for therapy using radiolabeled peptides is limited. The use of monoclonal antibodies (Mabs) may provide a more general solution as it is possible to generate high-affinity antibodies for a large variety of molecular targets. Such Mabs might provide specific delivery of cytotoxic radionuclides by molecular recognition of cancer-associated antigens on the surface of malignant cells. However, clinical studies on different types of human cancers revealed that the clinical effect of radioimmunotherapy (RIT) is limited to radiosensitive hematology malignancies. The breakthrough in RIT of more radioresistant solid tumors has not been achieved. Since the size of Mabs is relatively large (150 kDa), they remain in circulation for a long time, irradiating the radiosensitive bone marrow. Therefore, it is impossible to deliver a therapeutic dose to tumors without delivering unacceptably high doses to normal tissues [1,2]. One of the promising approaches to overcome these problems is to use engineered scaffold proteins (ESPs) for development of smaller molecules with high affinity for targeted radionuclide therapy.

Affibody^®^ molecules are one of the well-studied ESPs for radionuclide imaging and therapy. They are derived from a three-helix bundle scaffold, which is a small (molecular weight 7 kDa) and very stable protein domain, capable of exact refolding after thermal or chemical denaturing. Molecular display techniques enable selection of Affibody^®^ molecules binding to several targets with high specificity and affinity [3]. One of the examples of the successful application of Affibody^®^ technology for the development of targeting agents is the selection of a molecule capable of specific binding to human epidermal growth factor receptor 2 (HER2) [3]. HER2 is a member of the epidermal growth factor tyrosine kinase receptor family. Amplification or overexpression of HER2 has been documented in approximately 15–30% of breast cancers, 10–30% of gastric/gastroesophageal cancers, and 20–30% of ovarian cancers [4]. Previous studies showed that the Affibody^®^ molecule ZHER2:342 binds to the HER2 receptor with high affinity (dissociation constant at equilibrium, KD, ~22 pM) [5], which is crucial for high uptake and retention in the tumors. The size of Affibody molecules is small (~6.5 kDa) compared with the intact Mabs and Fab fragments (antigen-binding fragment, ~50 kDa). Because of their small size, high affinity, and specificity, Affibody^®^ molecules appeared as suitable ligands for both radionuclide imaging and therapeutic applications in both preclinical and clinical studies [6]. Since their size is below the cut-off for glomerular filtration (60 kDa), labeled Affibody^®^ molecules clear rapidly via the kidneys, which creates a precondition for an exquisite imaging contrast at the day of injection [7]. However, direct application of radiometal-labeled Affibody^®^ molecules for radionuclide therapy is complicated. After passing through the glomerular membrane, Affibody^®^ molecules are re-absorbed in the proximal tubuli of the kidney [8]. Rapid internalization by proximal tubuli cells results in the efficient retention of radiometals, leading to higher uptake in kidneys than in tumors [9]. The methods for reduction of renal uptake of radiopeptides, such as co- and pre-injection of positively charged amino acids or Gelofusine, turned out to be inefficient in the case of Affibody^®^ molecules [10]. Thus, a straightforward use of radiometal-labeled Affibody^®^ molecules for radionuclide therapy is not feasible. This reduces the opportunity to apply such a promising therapeutic radionuclide as lutetium-177.

Lutetium-177 (^177^Lu) [E_β−max_ = 497 keV, E_γ_ = 113 keV (6.4%), 208 keV (11%)] has a relatively long half-life (T_1/2_ = 6.73 days) and a maximum penetration of beta particles in tissue of 2 mm. The short range of beta particles makes them efficient in the treatment of small metastases. It is commercially available as a non-carrier added GMP-compliant product. The labelling chemistry of ^177^Lu is well established and permits stable labelling of targeting proteins and peptides [11]. Those properties make ^177^Lu one of the most clinically used radionuclides in targeted radionuclide therapy [1,12,13,14]. However, this nuclide has residualizing properties, which means that its retention time in kidneys would be long.

Previous studies demonstrated that one of the successful approaches for reduction of renal uptake is the fusion of Affibody^®^ molecules with a 5-kDa, non-cysteine-containing albumin-binding domain (ABD) [15]. This approach permits binding of the fusion protein to the serum albumin in the patient’s blood circulation, therefore increasing the retention time in blood and reducing the renal uptake [15,16]. The conjugate, ^177^Lu-CHX-A”-DTPA-ABD-(Z_HER2:342_)_2_, demonstrated a 25-fold reduction in renal uptake in mice in comparison with non-ABD-fused Affibody^®^ molecules [15]. Importantly, ABD-fused Affibody^®^ molecules are rescued from intracellular degradation by neonatal Fc receptor (FcRn) interaction, which increases their bioavailability [8]. The molecular weight of Affibody^®^-ABD-albumin adduct is about 77 kDa, which is appreciably less that weight of both intact IgG and (Fab’)_2_ fragment. The associated reduction of size provides an advantage for Affibody^®^-based constructs in extravasation and tumor penetration in comparison with immunoglobulins. In further studies, a second generation of ABD-fused Affibody^®^ molecules has been developed [17]. This targeting agent, Z_HER2:2891_-ABD-C-DOTA, contained a targeting part Z_HER2:2891_, which is based on the second-generation of Affibody^®^ scaffold [18]. The use of the new scaffold enables us to dramatically improve the thermal and chemical stability of Affibody^®^ molecules and permits their efficient peptide synthesis. An affinity matured ABD was placed at the C-terminus of Z_HER2:2891_. Both Affibody molecules and ABD do not contain cysteines. The introduction of a unique cysteine at the C-terminus of ABD enabled a site-specific conjugation of a maleimido derivative of the versatile chelator DOTA (acronym for dodecane tetraacetic acid, 1,4,7,10-tetraazacyclododecane-1,4,7,10-tetraacetic acid, other designation tetraxetan). The site-specificity of coupling allowed us to obtain conjugates with exactly defined structure and, therefore, reproducible biodistribution. This agent, denoted as ABY-027, was labeled with ^177^Lu, and its biodistribution has been evaluated. Animal studies demonstrated that ^177^Lu-ABY-027 provided further reduction of renal and hepatic uptake of radioactivity compared with the first generation targeting agent, ^177^Lu-CHX-A’’-DTPA-ABD-(Z_HER2:342_)_2_ [17]. Importantly, the tumor uptake of ^177^Lu-ABY-027 remained high and HER2-specific, which have been confirmed by an in vivo blocking experiment [17].

**Figure 1:**
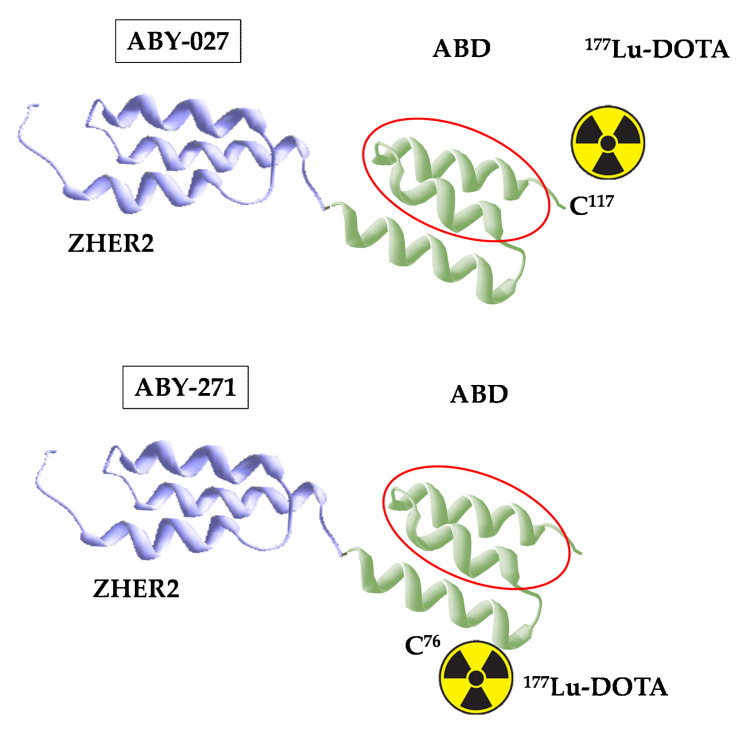
Structures of ^177^Lu-ABY-271 and ^177^Lu-ABY-027. The red line marks the segment of ABD where amino acids interacting with albumin are located.

Previous studies have demonstrated that label position in Affibody^®^ molecules has an appreciable impact on their biodistribution [19,20,21]. A systematic evaluation of constructs with a different position of the label enabled the selection of tumor-seeking probes with the best targeting properties. Structural and mutational analyses have demonstrated that amino acids on helix two and helix three of ABD are involved in binding of ABD to albumin, while helix one is essential for stabilization of the ABD structure [22,23]. Thus, positioning of a chelator on helix one would increase the distance between this chelator and the albumin binding site and, hopefully, might minimize the interference of a chelate with albumin binding. In this study, we evaluated a variant of Z_HER2:2891_-ABD, where the DOTA chelator is positioned on helix one on the back of ABD, which is opposite to helix two and three and responsible for binding to albumin. The goal was to test the hypothesis that such placement of the chelators would further reduce the steric interference of the label with the binding of the construct to albumin and provide labeling that would not modify the C-terminus of the polypeptide. For this purpose, a unique cysteine was introduced in position 76 of the Z_HER2:2891_-ABD fusion corresponding to the cysteine 14 position of the ABD molecule itself, as previously described [24], and the chelator was conjugated using maleimide chemistry to this cysteine to create a molecule with defined positioning of the DOTA chelator. Binding specificity, affinity, and cellular processing of the newly designed ^177^Lu-labeled anti-HER2 agent (further denoted ABY-271) (Figure 1), as well as its biodistribution and targeting properties, were evaluated in comparison with the properties of ABY-027 in the same batch of xenograft-bearing mice. To evaluate an effect of fusion with ABD-DOTA on renal uptake and residence in blood, the biodistribution of ^177^Lu-ABY-271 was compared with the biodistribution of^177^Lu-ABY-025 at 48 h. ABY-025 contains the same HER2-targeting Affibody^®^ molecule Z_HER2:2891_ as ABY-271 and conjugates to the same DOTA chelator using thiol-directed chemistry but is not fused with ABD.

## 2. Materials and Methods

### 2.1. General

Most of the chemicals used in the study were purchased from Sigma-Aldrich, Sweden AB (Stockholm, Sweden). Buffers used for labeling were prepared using high-quality Milli-Q water and purified from metal contaminations using Chelex 100 resin (Bio-Rad Laboratories, Hercules, CA, USA). No-carrier-added ^177^LuCl_3_ was purchased from Curium Pharma (Stockholm, Sweden). The NAP-5 size-exclusion columns were from GE Healthcare. Radioactivity was measured using an automated gamma-spectrometer with an NaI (TI) detector (2480 Wizard, Wallac, Finland). A Cyclone Storage Phosphor System and OptiQuant image analysis software (PerkinElmer, Waltham, MA, USA) were used for measuring the radioactivity distribution on the instant thin-layer chromatography (iTLC) strips. The Affibody^®^ molecules ABY-271, ABY-027 and ABY-025 were provided by Affibody AB, Solna, Sweden. The identity of probes was confirmed by mass-spectrometry. The purity of the constructs was over 95%, as determined by HPLC.

In vitro cell studies were performed using HER2-expressing ovarian cancer SKOV3 and breast cancer BT474 cells, both obtained from the American Type Culture Collection (ATCC, Manassas, MA, USA). Cells were cultured in Roswell Park Memorial Institute (RPMI) 1640 medium (Sigma-Aldrich, St. Louis, MO, USA), supplemented with 10% fetal calf serum, 2 mM L-glutamine, 100 IU/mL penicillin and 100 mg/mL streptomycin. Human Serum Albumin (HSA) was purchased from Sigma-Aldrich, Sweden AB (Stockholm, Sweden). Data on in vitro studies and biodistribution were analyzed by unpaired 2-tailed t-test and ANOVA using GraphPad Prism (version 9.00 for Windows; GraphPad Software LLC, San Diego, CA, USA) to determine significant differences.

### 2.2. Production of ABY-025, ABY-027 and ABY-271

The Affibody^®^ peptide ABY-027 was recombinantly produced using an *E. coli* process as earlier described [17]. ABY-025 was produced by solid phase peptide synthesis using the Fmoc/tBu strategy as earlier described [18].

ABY-271 [PEP41121] was produced by solid phase peptide synthesis using Fmoc/tBu. To ensure site-specific thiol-directed conjugation, a unique cysteine was coupled in position 76. The peptide was purified by preparative RP-HPLC and then conjugated in solution to maleimide-DOTA [CAS: 1006711-09-5] via residue Cys-76. The conjugated peptide was purified by preparative RP-HPLC using acetonitrile/water buffers containing TFA as the modifier. The purified peptide then underwent on-column salt exchange to convert from TFA to chloride counter-ion, and the final material lyophilized into 1 mg aliquots. The lyophilized molecules were stored at −20 °C before characterization and radio labelling. This work was performed as a fee for service by the contracting manufacturer Almac Sciences (Edinburgh, Scotland, UK) Ltd.

### 2.3. Characterization of ABY-027 and ABY-271

The characterization of ABY-027 and ABY-271 was performed to determine thermal stability, purity and isoelectric point (ABY-271) before performing biodistribution studies. The lyophilized molecules were dissolved in PBS containing 0.5 mM EDTA to a concentration of 1 mg/mL.

The molecules were characterized by Circular Dichroism (Jasco J-810 spectropolarimeter, Jasco Scandinavia AB) for reversibility of structure after heating to 90 °C, and with RP-UPLC (Agilent Zorbax 300 SB-C8 RRHD; 1.8 µm, 2.1 × 100 mm column). Elution by linear gradient of acetonitrile from 25–50% in 0.1% TFA during 8.3 min at a flow rate of 0.5 mL/min and at 40 °C. The correct molecular mass of the construct was confirmed using mass-spectrometry (API electrospray single quadrupole MSD, Agilent Technologies, Santa Clara, CA, USA). Isoelectric focusing was used to determine the isoelectric point for ABY-271 (Novex™ pH 3-10 IEF Protein Gels, ThermoFisher, Manassas, MA, USA).

### 2.4. Binding of ABY-027 and ABY-271 to HER2 and in the Presence of HSA or MSA

The binding kinetics of ABY-027 and ABY-271 to HER2, human serum albumin (HSA) and mouse serum albumin (MSA) were determined using Surface Plasmon Resonance (Biacore 8K, Cytiva, Uppsala, Sweden). Binding to HER2 by either ABY-027 or ABY-271 (10 nM) was performed in buffer or in the presence of HSA or MSA (100 nM). In these experiments, HER2 was immobilized to the chip (Series S Sensor Chip CM5, Cytiva) using amine coupling kit type 2 (Cytiva). Then, 10 mM sodium acetate pH 4.5 was used as an immobilization buffer.

### 2.5. Binding of ABY-027 and ABY-271 to HSA and MSA

The binding kinetics of ABY-027 and ABY-271 to HSA and MSA were determined using Surface Plasmon Resonance (Biacore 8K, Cytiva). In these experiments, HSA or MSA were immobilized to the chip (Series S Sensor Chip CM5, Cytiva) using amine coupling kit type 2 (Cytiva).

### 2.6. Labeling Chemistry

For the labeling of ABY-271, ABY-027 and ABY-025 with ^177^Lu, the same labeling protocol was used. Briefly, an aliquot of 30 µg of a protein in Milli-Q water was mixed with 20 µL of 0.2 M ascorbic acid in 0.2 M ammonium acetate, pH 5.8. A predetermined amount (37–70 MBq) of ^177^Lu was added, followed by vortexing. After incubation at 65 °C for 30 min, small (~1 µL) samples were taken for analysis of the radiochemical yield by iTLC. The ITLC strip was eluted with 0.2 M of citric acid, pH 2.0. The labeled conjugates were purified using NAP-5 columns, pre-equilibrated with PBS containing 1% BSA. The column was eluted with PBS. After purification, the purity was evaluated using iTLC. To validate iTLC, radio-HPLC of ^177^Lu-ABY-271 was performed. An Elite LaChrom system (Hitachi, VWR, Darmstadt, Germany) consisting of an L-2130 pump, a UV detector (L-2400), and a radiation flow detector (Bioscan, Washington, DC, USA) coupled in series was used. Analysis was performed using an analytical column (Phenomenex, Aschaffenburg, Germany; Luna^®^ 5 µm C18, 100 Å; 4.6 × 150 mm). HPLC conditions were as follows: A = 10 mM TFA/H2O; B =10 mM TFA/acetonitrile; UV-detection at 214 nm; gradient elution: 0–15 min at 5 to 70% B, 15–17 min at 70 to 95% B, 17–20 min at 5% B; and flow rate was 1.0 mL/min.

To evaluate label stability, aliquots of the fresh radiolabel was incubated with 500-fold molar excess of EDTA at 37 °C for 60 min. Incubation was also performed in PBS as a control. Samples were evaluated in triplicate. 

To evaluate stability in human serum albumin, freshly labeled ^177^Lu-ABY-271 and ^177^Lu-ABY-027 were mixed with a solution of protease-free human serum albumin (Merck, Kenilworth, NJ, USA) in PBS, pH 7.4, to obtain the final concentration of 50 mg/mL. This HSA concentration is close to the upper physiological level of albumin concentration in human blood. The solution was passed through a 0.22 µm sterile filter, and small aliquots were sealed in sterilized vials. The vials were incubated at 37 °C. At 1, 3, 24, 72 and 168 h after incubation started, three aliquots per sample were analyzed.

### 2.7. In Vitro Studies

The binding specificity of radiolabeled ABY-271 and ABY-027 to HER2-expressing cells was evaluated using SKOV-3 and BT-474 cells. Experiments were performed in triplicate. Approximately 7 × 10^5^ cells were seeded per well in 6-well plates the day before the experiment and were maintained in 2 mL complete RPMI 1640 medium at 5% CO_2_ and a 37 °C environment. For HER2 blocking, cells were incubated with a 1000-fold excess of non-labeled Affibody molecules for 30 min to saturate the receptors. Then, all cells were incubated with labeled conjugates (0.5 nM) for 60 min at 37 °C. After that, the media were collected, the cells were washed with 1 mL PBS, and the fractions were pooled. Thereafter, the cells were incubated with 1 mL 1 M NaOH at 37 °C for 20 min. The cell lysates were collected and washed with 1 mL NaOH followed by collecting to cell lysates. The radioactivity in cells and media was measured using an automatic gamma counter to calculate the percentage of cell-bound radioactivity. To evaluate and impact of HSA on binding specificity, this protein was added to the complete RPMI 1640 medium to obtain a concentration of 100 nM, and HSA-containing medium was used for the experiment, performed as described above.

The affinity of radiolabeled ABY-271 and ABY-027 binding to SKOV-3 cells was measured using LigandTracer (Ridgeview Instruments AB, Vänge, Sweden). SKOV-3 cells were seeded on the local area of a cell culture dish (NunclonTM, Size 100620, NUNC A/S, Roskilde, Denmark). In order to estimate the affinity properly, three concentrations of 0.25 nM, 0.75 nM and 1.25 nM of each radiolabeled conjugate were added in each affinity assay. The binding and dissociation kinetics were measured in real time as describe by Björke and Andwesson [25]. To investigate the influence of affinity with the presence of HSA, the experiment was performed in the presence of 100 nM HSA in complete RPMI 1640 medium instead of the complete medium. The data were analyzed by InteractionMap software (Ridgeview Diagnostics AB, Uppsala, Sweden) to calculate the dissociation constant at equilibrium (K_D_). The principle of data treatment using the InteractionMap is describe by Altshuh and co-workers [26].

Cellular processing and retention of ^177^Lu-ABY-271 and ^177^Lu-ABY-027 by HER2-expressing cells were studied in the presence of HSA in SKOV-3 and BT-474 cell lines. Approximately 7 × 10^5^ cells were seeded per dish in 3 cm petri dishes. A set of three dishes was used for each data point. Cells were incubated with a 5 nM solution of radiolabeled Affibody at 4 °C for 60 min. The medium with the labeled compound was removed, and the cells were washed with ice-cold PBS. Then, 1 mL complete medium containing 100 nM HSA was added to the cells, and further incubation was performed at 5% CO_2_ in a 37 °C environment. At pre-determined time points (1, 2, 4, 8 and 24 h), a group of three dishes was removed from the incubator, and the medium was collected. The cells were then washed with PBS and treated with 0.2 M glycine buffer containing 4 M urea, pH 2.0, for 5 min on ice. To calculate the membrane-associated activity, the cells were washed twice with 1 mL glycine buffer, the acidic fractions were collected, and their activity was measured. This activity was considered as membrane bound. After that, cells were incubated with 1 mL of 1 M NaOH at 37 °C for 20 min and collected with additional rinsing with 1 mL NaOH. The measured activity in alkaline fractions was considered as an internalized activity.

### 2.8. In Vivo Studies

The animal experiments were performed in accordance with national legislation on laboratory animal protection, and the study was approved by the local Ethics Committee for Animal Research in Uppsala (project identification code of approval 4/16, 26 February 2016). An overdosing of Rompun/Ketalar anesthesia was used for animal euthanasia.

HER2-positive and HER2-negative xenografts were established by subcutaneous injection of approximately 10^7^ SKOV-3 cells or Ramos cells, respectively, in the hind legs of female BALB/C *nu*/*nu* mice. In all biodistribution experiments, groups of 4 mice were used. The average animal weight was 18 ± 1.2 g at the time of the experiment. 

To evaluate the biodistribution of ^177^Lu-labeled ABY-271 in organs and tumors, and to obtain the data for calculations of dosimetry, 6 groups of mice with SKOV-3 xenografts were injected intravenously with ^177^Lu-ABY-271 in 100 µL of PBS. The injected activity was 260 kBq/mouse, and the injected protein was adjusted to 10 µg/mouse using non-labeled ABY-271. The average tumor weight was 0.19 ± 0.13 g. At 4, 24, 48, 72,168, and 336 h after injection, one group of mice was euthanized by injection of overdosing anesthesia followed by heart puncture. The blood, heart, lung, liver, spleen, pancreas, stomach wall, small intestine wall, kidney, tumor, muscle, bone, brain, stomach content, small intestine, large intestine, and the remaining carcass were collected. Organs and tissue samples were weighed, and their activity was measured. Organs and tumor uptake were calculated as the percentage of the injected dose per gram of the sample (% ID/g), while for intestines and carcass uptake, the percentage of injected dose (% ID) per whole sample was calculated. 

To test in vivo specificity, 1 group of mice with Ramos xenografts were injected with ^177^Lu-ABY-271 (10 µg in 100 µL of PBS). The average Ramos tumor weight was 0.26 ± 0.24 g. The same organs as in the groups for ^177^Lu-ABY-271 and the tumors were collected and measured at 48 h.

To evaluate the effect of fusion of Z_HER2:2891_ with ABD on biodistribution, 1 group of mice with SKOV-3 xenografts were injected with 260 kBq of ^177^Lu-ABY-025 (5 µg in 100 µL). The average tumor weight was 0.06 ± 0.02 g. The same organs as in the group of ^177^Lu-ABY-271 and the tumor were collected and measured at 48 h.

To compare the biodistribution of ^177^Lu-ABY-027 with ^177^Lu-ABY-271, 2 groups of mice with SKOV-3 xenografts were injected with 260 kBq of ^177^Lu-ABY-027 (10 µg) in 100 µL PBS. The average tumor weight was 0.26 ± 0.22 g. The blood, heart, lung, liver, spleen, pancreas, kidney, tumor, muscle, bone, gastro-intestine and remaining carcass were collected, and the biodistribution was measured at 48 and 168 h.

In vivo imaging was performed to obtain a visual confirmation of the biodistribution data. Two mice with Ramos xenografts and two mice with SKOV-3 xenografts were injected with 7.2 MBq (10 µg) of ^177^Lu-ABY-271, and two mice with SKOV-3 xenografts were injected with either 8.2 MBq (10 µg) of ^177^Lu-ABY-027 or 3.9 MBq (5 µg) of ^177^Lu-ABY-025. All labeled conjugates were injected 48 h before the imaging experiment. Imaging was performed using nanoScan SPECT/CT (Mediso Medical Imaging Systems, Budapest, Hungary). The animals were anesthetized using sevoflurane and placed in a prone position on a warmed scanner bed. Anesthesia was maintained throughout the study using 1.5–2.0% sevoflurane in 50% oxygen and 50% medical air (flow of 0.5 L/min). CT scans were acquired using the following parameters: X-ray energy peak of 50 keV; 670 µA; 480 projections; and 5.26 min acquisition time. SPECT acquisition was performed at the following parameters: energy windows 103–124 and 188–230 keV, matrix of 256 × 256, and acquisition time of 20 min. SPECT raw data were reconstructed using Tera-Tomo™ 3D SPECT reconstruction technology (version 3.00.020.000; Mediso Medical Imaging Systems Ltd., Budapest, Hungary): normal dynamic range; 30 iterations; and one subset. CT data were reconstructed using Filter Back Projection in Nucline 2.03 Software (Mediso Medical Imaging Systems Ltd., Budapest, Hungary). SPECT and CT files were fused using Nucline 2.03 Software and are presented as maximum intensity projections in the RGB colour scale.

## 3. Results

### 3.1. Production and Characterization of ABY-271 and ABY-027

ABY-271 was successfully synthesized by the fee for service contractor Almac (Edinburgh, Scotland, UK). The correct molecular mass of the construct (12,494 Da) was confirmed using mass-spectrometry, and the purity by RP-UPLC was determined as 95% by the service contractor. ABY-027 was recombinantly produced in *E.coli* as earlier described [15]. The molecular mass was determined to be correct (12650 Da), and the purity was 95%. The isoelectric point for ABY-271 was determined to secure compatibility with the chosen buffer during labelling. The pI was 5.4.

Measurement by Circular dichroism showed that ABY-271 and ABY-027 had a typical alpha helical structure. The CD spectra before and after heating to 90 °C coincided, which showed that the proteins were correctly refolded.

### 3.2. Affinity Measurements Using SPR

The affinity of both ABY-271 and ABY-027 to HSA was extremely high, but exact quantitate assessment was difficult to perform because of very slow off-rate (Table 1). The data for MSA were more reliable. Surprisingly, the affinity of ABY-271 to MSA was approximately twice as low compared with the affinity of ABY-027. The affinity of both variants to human HER2 was comparable in all conditions, but ABY-027 always had lower values. Addition of 100 nM of MSA or HSA reduced the affinity of both variants to HER2 by approximately one order of magnitude. 

### 3.3. Labeling Chemistry

**Figure 2:**
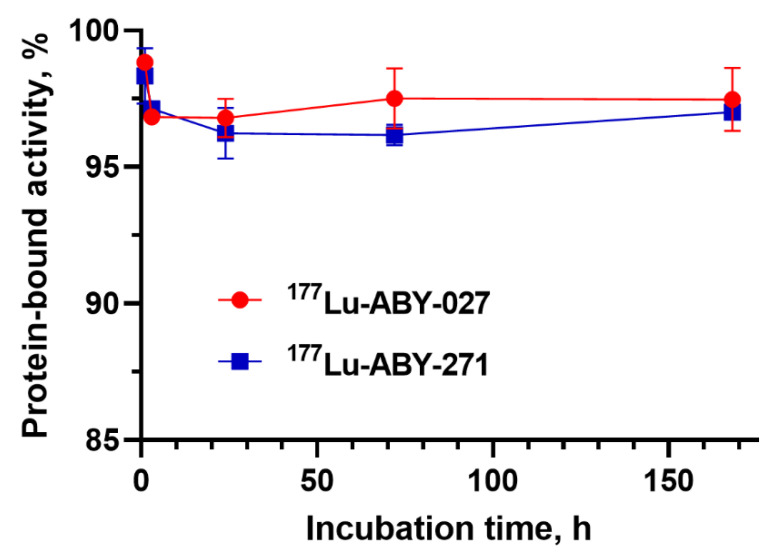
Protein-bound activity of ^177^Lu-ABY-271 and ^177^Lu-ABY-027 during incubation with HSA at 37 °C. The data are presented as an average value from 3 samples ± SD.

The labeling of ABY-271 was rapid and efficient, providing a yield of 95.9 ± 2.9% after 30 min of incubation. Purification using NAP-5 columns provided radiochemical purity of 100 ± 0%. After incubation with a 500-fold molar excess of EDTA for 1 h, there was no measurable release of radioactivity. Comparison of ^177^Lu-ABY-271 and ^177^Lu-ABY-027 stability in a solution of HSA in PBS pH 7.4 is presented in Figure 2. The data suggest a minimal release (if any) of low molecular weight of ^177^Lu from proteins. To validate radio-ITLC, radio-HPLC of ^177^Lu-ABY-271 was performed, and it demonstrated that no fragmentation occurred after labeling and purification. The yield of labeling of ABY-027 and ABY-025 was 97.8 ± 0.1% and 98.8%, respectively.

### 3.4. In Vitro Studies

**Figure 3:**
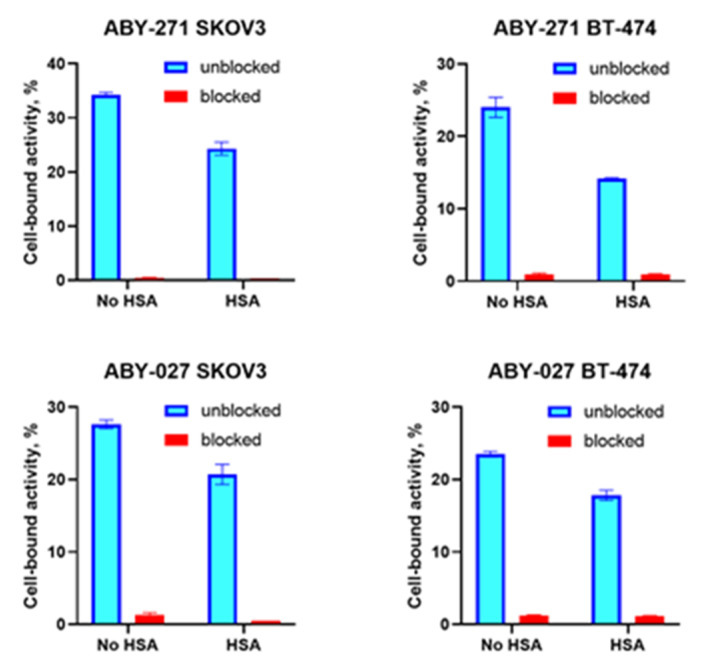
In vitro specificity test for ^177^Lu-ABY-271 and ^177^Lu-ABY-027 on SKOV-3 and BT-474 cells in the absence and in the presence of HSA. The data are presented as an average value from 3 samples ± SD.

The results of the in vitro binding specificity of ^177^Lu-ABY-271 and ^177^Lu-ABY-027 are shown in Figure 3. Binding of ^177^Lu-ABY-271 and ^177^Lu-ABY-027 to HER2-expressing SKOV-3 and BT-474 cells was significantly lower (*p* < 0.001) after saturation of HER2 receptors in blocked groups both in the presence and absence of HSA. Significantly lower (*p* < 0.005) uptake of both ABY-271 and ABY-027 was observed in the presence of HSA, which indicated a lower affinity.

**Figure 4:**
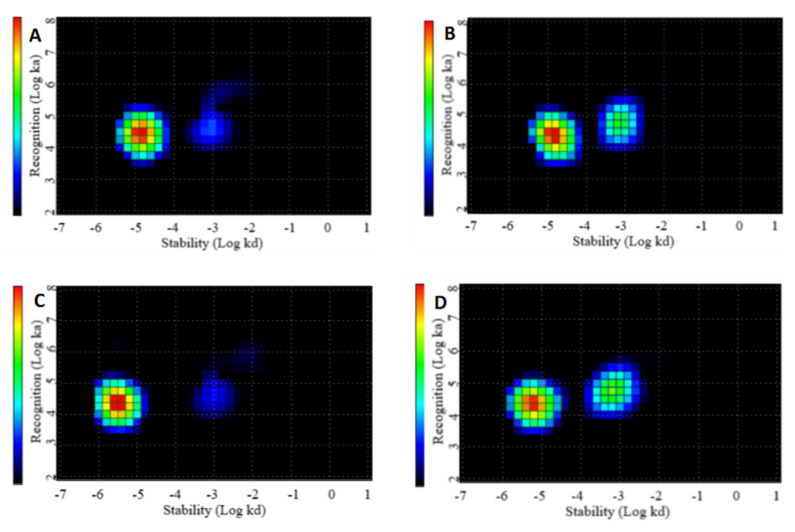
InteractionMap of (**A**) ^177^Lu-ABY-271 without HSA, (**B**) ^177^Lu-ABY-271 with HSA, (**C**) ^177^Lu-ABY-027 without HSA and (**D**) ^177^Lu-ABY-027 with HSA binding to HER2-expressing SKOV3 cells. Binding was measured at three concentrations of 0.25 nM, 0.75 nM and 1.25 nM.

The affinities of ^177^Lu-labeled ABY-271 and ABY-027 binding to HER2-expressing SKOV-3 cells (both in the absence and presence of HSA) were evaluated by InteractionMap analysis of the LigandTracer sensorgrams. The data are presented in Table 2 and in Figure 4.

All variants had comparable association rates (in the range of 0.6 × 10^5^ to 1.1 × 10^5^ M^−1^s^−1^) but differed in dissociation rates. Typically, two kinds of interaction were observed for each construct, one with subnanomolar affinity and another in the range of 10–30 nM. Adding of HSA resulted in a decrease in affinity for both ^177^Lu-ABY-271 and ^177^Lu-ABY-027. Interestingly, the affinity of ^177^Lu-ABY-271 binding to living cells was higher than the affinity of ^177^Lu-ABY-027.

**Figure 5:**
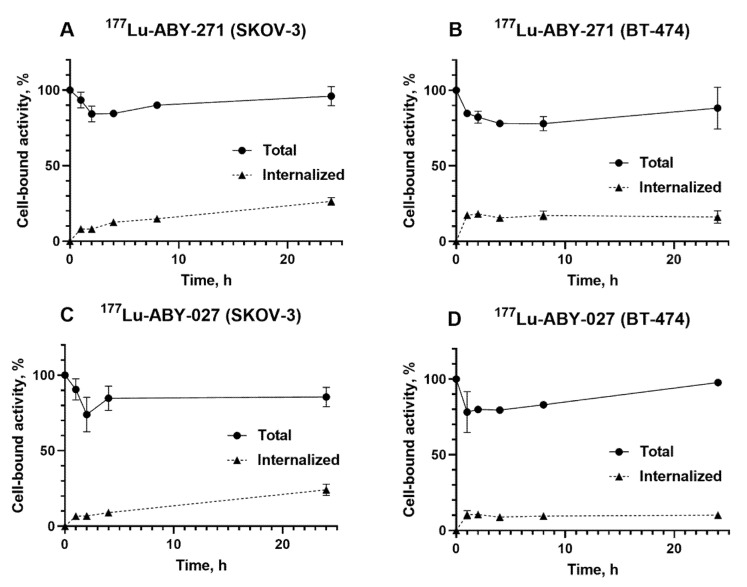
Cellular processing of ^177^Lu-ABY-271 (**A**,**B**) and ^177^Lu-ABY-027 (**C**,**D**) on SKOV-3 and BT-474 in the presence of HSA after interrupted incubation (average of 3 samples ± SD).

The cellular retention profile (Figure 5) showed that the retention of cell-associated activity was good for both ^177^Lu-ABY-271 and ^177^Lu-ABY-027. In both cases, the cell-associated activity was over 85% of initially bound after 24-h-long incubation in the presence of HSA (no significant difference (*p* > 0.05) in one-way ANOVA analysis with Bonferroni test for multiple comparison). The pattern was similar for both SKOV-3 and BT-474 cells, with quite slow internalization of both radiolabeled constructs. However, the internalized activity was somewhat higher for SKOV-3 cells compared with BT-474 (adjusted *p* value of less than 0.05 for ^177^Lu-ABY-271 and less than 0.005 for ^177^Lu-ABY-027 in one-way ANOVA analysis with Bonferroni test for multiple comparison).

### 3.5. In Vivo Studies

**Figure 6:**
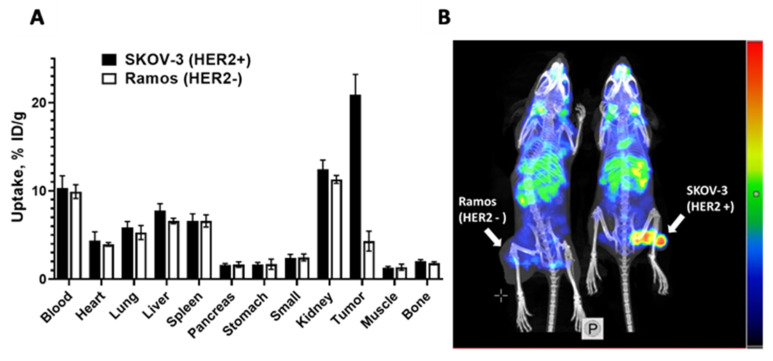
The in vivo binding specificity of ^177^Lu-ABY-271 was evaluated by comparison of the uptake in HER2-positive SKOV-3 and HER2-negative Ramos xenografts in BALB/C *nu*/*nu* mice 48 h after injection. (**A**) Biodistribution. Results of ex vivo measurements are presented as % ID/g ± SD (*n* = 4). (**B**) Imaging of ^177^Lu-ABY-271 in BALB/C *nu*/*nu* mice bearing SKOV-3 and Ramos xenografts 48 h after injection (linear scale, max. 1800 kBq/mL).

Results from the in vivo specificity test showed that the uptake of ^177^Lu-ABY-271 in HER2-expressing SKOV-3 xenografts in BALB/C *nu*/*nu* mice was 5-fold (*p* < 0.0005) higher than that in HER2-negative Ramos xenografts, while the accumulation of radioactivity in other organs was the same (Figure 6A). This indicates that the in vivo accumulation of ^177^Lu-ABY-271 is highly HER2-specific. The results were confirmed by the imaging performed 48 h after injection of ^177^Lu-ABY-271 both in SKOV-3 and Ramos xenografts (Figure 6B).

**Figure 7:**
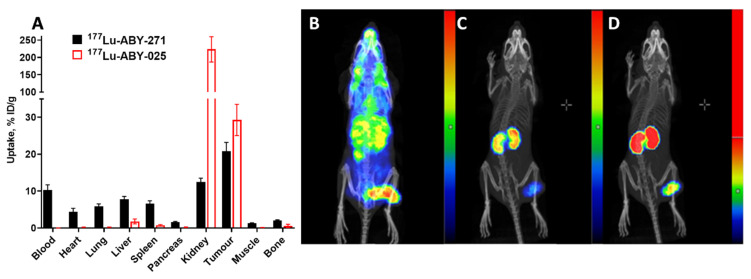
(**A**) Uptake of radioactivity 48 h after injection of ^177^Lu-ABY-271 and ^177^Lu-ABY-025 in SKOV-3 xenograft-bearing mice (average of 4, % ID/g ± SD). (**B**) Imaging of distribution of ^177^Lu-ABY-271 (linear scale, max. 1800 kBq/mL). (**C**) Imaging of distribution of ^177^Lu-ABY-025 (full scale, max. 7900 kBq/mL). (**D**) Imaging of distribution of ^177^Lu-ABY-025 (the scale is adjusted to the first red pixels in the tumor, mark at 3500 kBq/mL).

To evaluate the effect of fusion with ABD, the biodistribution of ^177^Lu-ABY-271 and ^177^Lu-ABY-025 (containing the same targeting Z_HER2:2891_ as ABY-271 but not fused with ABD) was compared in BALB/C *nu*/*nu* mice bearing HER2-expressing SKOV-3 xenograft 48 h after injection. The data are presented in Figure 7A. The activity uptake of ^177^Lu-ABY-271 in the kidney was reduced 18-fold in comparison with the uptake of ^177^Lu-ABY-025. The activity concentration of ^177^Lu-ABY-271 in blood remained 450-fold higher 48 h after injection compared with activity of ^177^Lu-ABY-025. The imaging of distribution of ^177^Lu-ABY-271 and ^177^Lu-ABY-025 48 h after injection (Figure 7B–D) confirmed the biodistribution data and demonstrated that the retention of ^177^Lu-ABY-271 in blood is longer than that of ^177^Lu-ABY-025, but the renal uptake of ^177^Lu-ABY-271 was much lower compared with the uptake of ^177^Lu-ABY-025.

Data concerning the biodistribution of ^177^Lu-ABY-271 in BALB/C *nu*/*nu* mice bearing HER2-expressing SKOV-3 xenografts at different time points after injection are shown in Table 3. A slow clearance of ^177^Lu-ABY-271 from the blood was observed, with a biological half-life of 28 h. Clearance of ^177^Lu-ABY-271 from normal organs and tissues followed clearance from the blood. The tumor uptake of ^177^Lu-ABY-271 was already higher than the radioactivity concentration in blood and all other organs and tissues at the 24-h time point after injection. The uptake of ^177^Lu-ABY-271 in the tumor peaked 72 h after injection, followed by slow washout. 

**Figure 8:**
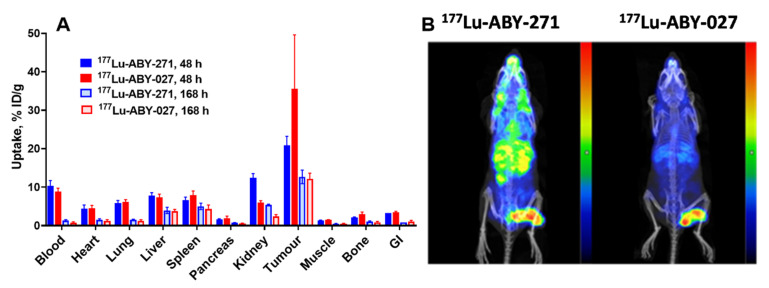
(**A**) Comparison of the biodistribution of ^177^Lu-ABY-271 and ^177^Lu-ABY-027 in HER2-expressing SKOV-3 xenograft-bearing BALB/C *nu*/*nu* mice 48 and 168 h after injection. (**B**) Imaging of SKOV-3 xenograft-bearing mice injected with ^177^Lu-ABY-271 (linear scale, max. 1800 kBq/mL) and ^177^Lu-ABY-027 (linear scale, max. 3000 kBq/mL) 48 h after injection.

Results of the comparison of the biodistribution of ^177^Lu-ABY-271 and ^177^Lu-ABY-027 in HER2-expressing SKOV-3 xenograft-bearing BALB/C *nu*/*nu* mice 48 and 168 h after injection are presented in Figure 8. At 48 h after injection, the tumor uptake of ^177^Lu-ABY-271 was significantly lower (*p* < 0.005) than that of ^177^Lu-ABY-027, while the renal uptake of ^177^Lu-ABY-271 was higher (*p* < 0.0001) than ^177^Lu-ABY-027. At 168 h after injection, the uptake of ^177^Lu-ABY-271 in the kidney was significantly higher (*p* < 0.0001) than that of ^177^Lu-ABY-027, while the uptake of ^177^Lu-ABY-271 and ^177^Lu-ABY-027 in the tumor were the same.

## 4. Discussion

The binding of small targeting proteins to albumin is a promising approach to modifying their clearance rate from blood and modulating biodistribution in a desirable way [16]. Direct chemical coupling of Affibody molecules to albumin has been evaluated by Hoppmann and co-workers [27]. The authors demonstrated that the maximum tumor uptake (~14% ID/g between 24 and 48 h) exceeded the renal uptake (4.17 ± 1.01% ID/g at 24 h). However, the hepatic uptake was approximately equal to the tumor uptake (12.92 ± 2.4% ID/g at 24 h). This excludes the use of such a construct for radionuclide therapy. One of major issues in such an approach is poor control over the conjugation process. The authors reported that chemical conjugation resulted in constructs containing one to five Affibody molecules and around two DOTA chelators per albumin. Apparently, this approach would be associated with overwhelming regulatory issues due to uncertainty with the structure of the resulting therapeutic. 

The use of fusion or site-specific conjugation of Affibody molecules to ABD permits us to perform binding to albumin in a more controllable way. The structure of the resulting constructs is well defined. This allows for evaluation of the impact of different modifications on targeting properties. In the previous study, it was demonstrated that as an ABD fusion protein, ABY-027 labeled with ^177^Lu has appropriate biodistribution in the murine model, suggesting that it is potentially suitable for radionuclide therapy [17]. It would be logical to position the DOTA via a site-specific maleimide chemistry conjugation to a unique cysteine (C14) on helix one on the back of the ABD molecule, away from the albumin binding site but with no linker and thus a very tight hydrodynamic volume. In this study, the resulting newly designed ABD fusion Affibody molecule, ABY-271, was evaluated, with the DOTA site specifically conjugated to position C76 (corresponding to C14 on ABD itself). The comparison with ^177^Lu-ABY-027 was performed in the same batch of mice bearing HER2 expressing xenografts to minimize the batch-to-batch variability of animal physiology and passage-dependent features of cell lines. Change of the chelator position had an obvious effect on targeting properties. 

The labeling of ABY-271 with ^177^Lu was efficient (radiochemical over 95%) and stable, both under the EDTA challenge and during incubation with albumin solution, with concentration mimicking its concentration in human serum (Figure 2). In vitro binding specificity assay has shown that the binding of ^177^Lu-ABY-271 and ^177^Lu-ABY-027 to HER2-expressing cells are specific both in the presence and absence of HSA (Figure 3). The addition of albumin resulted in the reduction of binding to cells, which is in agreement with the reduction of affinity to HER2 after the adding of albumin, observed in SPR measurements, mainly due to decreased association rate. Furthermore, LigandTracer measurements of binding to living HER2-expressing cells also demonstrated a decrease in ^177^Lu-ABY-271 and ^177^Lu-ABY-027 affinity to HER2 in the presence of 100 nM albumin (Table 2 and Figure 4). The effect of adding albumin was stronger for ^177^Lu-ABY-271 (2.5-fold decrease) than for ^177^Lu-ABY-027 (~1.3-fold). Still, the affinity was in a low nanomolar range, which should be sufficient for efficient targeting using a protein with the size of an Affibody-ABD-albumin adduct according to mathematical modelling [28].

Cellular processing and retention in the presence of HSA had a similar pattern for both variants (Figure 5). The majority of cell-associated activity was bound to the membrane throughout the observation time, and internalization of the radiolabeled compound was slow (26% in SKOV-3 and 16% in BT-474 after 24 h of interrupted incubation). This pattern is an agreement with previous data for ^177^Lu-ABY-027 measured without albumin [17]. Thus, the processing pattern of ABD-fused Affibody molecules is reproducible, independent of the presence of albumin, and dependent on a malignant cell line origin only to a limited extent. 

The results of the in vivo study confirm that the newly designed ^177^Lu-ABY-271 specifically targets HER2-expressing SKOV-3 xenografts, as the tumor uptake was significantly (*p* < 0.0005) higher than the uptake in HER2-negative Ramos xenografts (Figure 6). It has to be noted that the observed uptake of radiolabeled ABY-271 in Ramos xenografts is most likely due to the enhanced permeability and retention (EPR) effect [29] caused by binding to bulky albumin. The comparison of the biodistribution of ^177^Lu-ABY-271 and ^177^Lu-ABY-025 (Figure 7A) demonstrated that fusion with ABD increases retention in the blood and the tumor-to-kidney ratio of the radiolabeled conjugate. This was confirmed by the imaging of the biodistribution of ^177^Lu-ABY-271 and ^177^Lu-ABY-025 48 h after injection (Figure 7B). As expected, the elimination half-life of the parental Z_HER2:2891_ Affibody molecule from blood increased more than 50-fold from 0.5 h [30] to 28 h (Table 2). The tumor uptake of ^177^Lu-ABY-271 24 h and afterwards was higher than the uptake in any other organ (Table 2). However, the tumor-to-kidney ratio was lower than three at any time point.

Comparison of the biodistribution of ^177^Lu-ABY-271 and ^177^Lu-ABY-027 demonstrates that ^177^Lu-ABY-271 provides a lower tumor-to-kidney ratio than ^177^Lu-ABY-027 (Figure 8). This indicates that ^177^Lu-ABY-271 is not as efficient as ^177^Lu-ABY-027 for the delivery of high-absorbed doses to tumors while avoiding high doses to the critical organ, the kidney. The most important lesson from this comparison is that the position of DOTA on the ABD domain impacts the biodistribution of ^177^Lu-labeled Affibody molecules. 

The major remaining question is why position has such an influence. ABY-027 had higher affinity to MSA, but the difference was only two-fold (Table 1). Albumin is an abundant protein in the blood (~0.3 mM in mice [31], and the major part of a binder population with subnanomolar affinity should be bound to albumin. A possible insight might be gained from studies on the influence of the position of the DOTA chelator on Affibody molecules [20,21]. In these studies, similar placement of a chelator on a helix opposite to the binding site did not influence affinity (measured by SPR) but resulted in a noticeable decrease in tumor uptake. Apparently, the affinity measurements in vitro do not provide sufficient information for prediction of in-vivo properties. It is possible that the positioning of a chelator on a helix destabilizes the protein structure in vivo. Additionally, a therapeutic targeted protein undergoes multiple off-target interactions in a living body, which is difficult to account for and model in vitro. Furthermore, all elements of a construct participate in such interactions, and the character and strength of these interactions depend on their mutual position. For example, there was no difference in the affinity of HER3-binding ABD-binding constructs to albumin [32]. However, the renal uptake between constructs with the Affibody molecule on the C-terminus of ABD and N-terminus differed four-fold. One cannot exclude the fact that shielding of the C-terminal part of ABD by conjugation to a hydrophilic DOTA-molecule plays an important role in reducing kidney uptake, potentially by reduction of scavenger receptor uptake in the proximal tubuli. A strong influence of the valency of the targeting part and hydrophilicity of a linker to cytotoxic payload affects the biodistribution pattern and renal uptake of Affibody-ABD conjugates with maytansine [33]. 

Affinity Affibody-ABD fusions to cancer-associated antigen and albumin, as well as their biodistribution, are influenced by the position where a chelator is coupled. Potentially, this might help to improve targeting properties through the selection of an optimal position. However, data from this study show that the targeting properties might also be compromised by suboptimal chelator placement. The common-sense approach of “the bigger distance of a chelator from binding site of ABD reduces interference with binding to albumin resulting lower renal uptake” does not work. Systematic studies are required to obtain a good understanding of the structure–properties relationship and design successful targeting agents.

## 5. Conclusions

The fusion protein ABY-271 provides HER2-specific binding and suitable biodistribution for therapy. Fusion with ABD results in a reduction of the renal uptake of ^177^Lu-ABY-271, as compared with the targeting Affibody molecule alone (ABY-025), and an increase of retention in blood in BALB/C *nu*/*nu* mice bearing SKOV3 xenografts. However, ^177^Lu-ABY-271 is not as efficient as ^177^Lu-ABY-027 for delivering high doses to tumors while sparing kidneys. Even a small modification, such as the position of DOTA on the ABD domain, has a substantial impact on the properties of the biodistribution of radiometal-labeled Affibody molecules.

## Figures and Tables

**Table 1:** Equilibrium dissociation constants (K_D_) of binding ABY-027 and ABY-271 to human serum albumin (HSA), mouse serum albumin (MSA) and HER2.

Target	Equilibrium Dissociation Constant (K_D_), M	
	ABY-271	ABY-027
HSA	(4.4 ± 3.5) × 10^−15^	(9.5 ± 5.9) × 10^−15^
MSA	(4.8 ± 0.5) × 10^−11^	(2.46 ± 0.08) × 10^−11^
HER2 (no albumin)	(4 ± 1) × 10^−10^	(1.3 ± 0.1) × 10^−10^
HER2 in the presence of 100 nM HSA	(3 ± 2) × 10^−9^	(2.8 ± 0.6) × 10^−9^
HER2 in the presence of 100 nM MSA	(8.1 ± 0.2) × 10^−9^	(3.9 ± 0.8) × 10^−9^

**Table 2:** InteractionMap evaluation of the affinity of ^177^Lu labeled ABY-271 and ABY-027 binding to HER2-expressing SKOV3 cells in the presence and absence of HSA.

Testing Conditions	K_D1_ (pM)	K_D2_ (nM)	Header
^177^Lu-ABY-271 + HSA	321 ± 66	13.3 ± 2.9 (29–39%)	(*n* = 5)
^177^Lu-ABY-271 no HSA	131 ± 31	24.3 ± 2.7 (10–12%)	(*n* = 5)
^177^Lu-ABY-027 + HSA	674 ± 29	11.5 ± 0.7 (28%)	(*n* = 2)
^177^Lu-ABY-027 no HSA	516 ± 47	17.0 ± 4.6 (15%)	(*n* = 2)

**Table 3:** Biodistribution of ^177^Lu-ABY-271 in BALB/C *nu*/*nu* mice bearing SKOV-3 xenografts. Uptake is expressed as % ID/g, which is corrected for decay and presented as average value from 4 mice ± SD.

Organ	Uptake					
	4 h	24 h	48 h	72 h	168 h	336 h
Blood	25.2 ± 1.0	15.9 ± 1.5	10.3 ± 1.4	6.3 ± 0.6	1.3 ± 0.3	0.13 ± 0.03
Heart	6.6 ± 1.1	5.0 ± 0.7	4.4 ± 1.0	3.3 ± 0.1	1.5 ± 0.3	0.49 ± 0.07
Lung	9.2 ± 0.3	7.5 ± 0.7	5.9 ± 0.6	4.5 ± 0.4	1.5 ± 0.2	0.33 ± 0.09
Liver	5.8 ± 0.5	6.9 ± 1.0	7.8 ± 0.8	7.1 ± 0.8	3.9 ± 0.8	1.52 ± 0.28
Spleen	4.8 ± 0.4	6.0 ± 0.9	6.6 ± 0.8	6.2 ± 0.4	5.0 ± 0.8	3.09 ± 0.70
Pancreas	1.7 ± 0.2	2.1 ± 0.3	1.6 ± 0.2	1.4 ± 0.3	0.7 ± 0.1	0.19 ± 0.03
Stomach wall	2.0 ± 0.4	1.9 ± 0.2	1.6 ± 0.2	1.2 ± 0.1	0.4 ± 0.08	0.11 ± 0.02
Small intestine wall	3.8 ± 0.9	3.0 ± 0.6	2.4 ± 0.4	2.1 ± 0.3	0.8 ± 0.2	0.25 ± 0.04
Kidney	15.1 ± 0.8	14.4 ± 1.0	12.5 ± 1.0	10.2 ± 0.7	5.4 ± 0.2	1.86 ± 0.16
Tumor	8.0 ± 3.7	18.2 ± 3.9	20.9 ± 2.3	24.2 ± 5.9	12.7 ± 1.8	2.46 ± 0.16
Muscle	1.4 ± 0.2	1.6 ± 0.2	1.3 ± 0.1	1.0 ± 0.1	0.5 ± 0.09	0.14 ± 0.02
Bone	2.2 ± 0.3	2.3 ± 0.1	2.1 ± 0.2	1.8 ± 0.4	1.0 ± 0.2	0.45 ± 0.12
Brain	0.5 ± 0.1	0.3 ± 0.03	0.3 ± 0.05	0.2 ± 0.05	0.06 ± 0.009	0.02 ± 0.011
Stomach content *	0.1 ± 0.1	0.07±0.02	0.04±0.01	0.04 ± 0.05	0.008 ± 0.007	0.002 ± 0.004
Small intestine with content *	3.1 ± 0.2	2.1 ± 0.3	1.7 ± 0.2	1.2 ± 0.2	0.5 ± 0.14	0.12 ± 0.06
Large intestine with content *	1.1 ± 0.4	1.7 ± 0.3	1.6 ± 0.6	1.1 ± 0.4	0.4 ± 0.07	0.10 ± 0.03

* Data for stomach content, intestines with content and carcass are presented as % ID per whole sample.

## Data Availability

Data is contained within the article.
